# Correction: The pyruvate dehydrogenase complex regulates mitophagic trafficking and protein phosphorylation

**DOI:** 10.26508/lsa.202402684

**Published:** 2024-03-14

**Authors:** Panagiota Kolitsida, Vladimir Nolic, Jianwen Zhou, Michael Stumpe, Natalie M Niemi, Jörn Dengjel, Hagai Abeliovich

**Affiliations:** 1 Department of Biochemistry, Food Science and Nutrition, Hebrew University of Jerusalem , Rehovot, Israel; 2 Department of Biology, University of Fribourg , Fribourg, Switzerland; 3 Department of Biochemistry and Molecular Biophysics, Washington University , St. Louis, MO, USA

## Abstract

Mutations in the PDC affect the phosphorylation and mitophagic trafficking of matrix proteins, through the novel regulation of associated kinases and a phosphatase. We suggest that this occurs by the direct allosteric regulation of the phosphatase and kinases by the PDC.

Article: Kolitsida P, Nolic V, Zhou J, Stumpe M, Niemi NM, Dengjel J, Abeliovich H (2023 Jul 13) The pyruvate dehydrogenase complex regulates mitophagic trafficking and protein phosphorylation. Life Sci Alliance 6(9): e202302149. doi: 10.26508/lsa.202302149. PMID: 37442609.

The authors would like to correct an error in Fig 6. A minus sign appears instead of a plus sign for *Aup1-HA* in the fourth column of the figure, which renders the results meaningless. The corrected version of Fig 6 is now provided.

**Figure:**
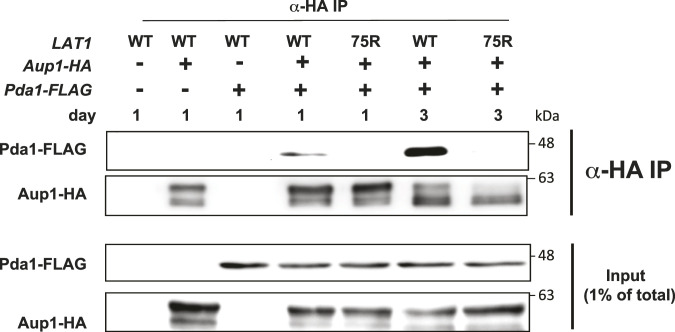


## Supplementary Material

Reviewer comments

