# Supplementary material for: The pyruvate dehydrogenase complex regulates mitophagic trafficking and protein phosphorylation
Source: Life Sci Alliance. 2023 Jul 13;6(9):e202302149. doi: 10.26508/lsa.202302149 (PMC10345312; doi:10.26508/lsa.202302149)
Supplement: Supplementary file 2 [file LSA-2023-02149_TableS2.doc]

**Supplementary Table 2. Yeast Strains used in this study**

| **Strain** | **Genotype** | **Reference/source** |
| --- | --- | --- |
| ***Saccharomyces cerevisiae* strains** | | |
| **HAY75**  Wild type, progeny of SEY 6211 and SEY6210 | *MATα leu2-3,112 ura3-52 his3-Δ200 trp1-Δ901 lys2-801 suc2-Δ9* | (Abeliovich et al. *Mol. Biol. Cell*, 2003) |
| **PKY832** | HAY75, *pda1Δ::HIS5 S.p.* | This study |
| **HAY1239** | HAY75, *atg32Δ::G418^R^* | (Abeliovich et al., *Nat Commun,*  2013) |
| **TVY1** | HAY75*, pep4Δ::LEU2* | (Gerhardt et al., *J. Biol. Chem,* 1998) |
| **PKY334** | HAY1239, MDH1-GFP:: *HIS5 S.p* | Kolitsida et al, *PNAS,* 2019 |
| **PKY2046** | HAY75, ACO2-GFP::*G418 ^R^* | This study |
| **PKY2047** | HAY75, ACO1-GFP::*G418 ^R^* | This study |
| **PKY2045** | HAY75, IDP1-GFP::*G418 ^R^* | This study |
| **PKY973** | HAY75, QCR2-GFP::*G418 ^R^* | This study |
| **PKY974** | HAY75, MDH1-GFP::*G418 ^R^* | This study |
| **PKY2041** | PKY832, ACO2-GFP::*G418 ^R^* | This study |
| **PKY2041** | PKY832, QCR2-GFP::*G418 ^R^* | This study |
| **PKY2023** | PKY832, MDH1-GFP::*G418 ^R^* | This study |
| **PKY2044** | PKY832, IDP1-GFP::*G418 ^R^* | This study |
| **PKY2063** | PKY832, ACO1-GFP::*G418 ^R^* | This study |
| **PKY2089** | HAY75, *mpc1Δ::HIS5 S.p.* | This study |
| **PKY2104** | PKY2089, MDH1-GFP::*G418 ^R^* | This study |
| **PKY1089** | TVY1, MDH1 GFP::*G418 ^R^* | This study |
| **PKY1093** | PKY1089:: *pda1Δ::HIS5 S.p* | This study |
| **PKY2202** | HAY75*, pep4Δ::*NAT*^R^* | This study |
| **PKY2206** | PKY2202, *pda1Δ::HIS5 S.p* | This study |
| **PKY2211** | PKY2206, MDH1-GFP::*G418 ^R^* | This study |
| **PKY2205** | PKY2202, MDH1-GFP::*G418 ^R^* | This study |
| **PKY2216** | PKY2205, pCU305::*LEU*, pCU306::*URA3* | This study |
| **PKY2219** | PKY2211, pCU305::*LEU*, pCU306::*URA3* | This study |
| **PKY2220** | PKY2211, PKP1 pCU305::*LEU*, PKP2 pCU306::*URA3* | This study |
| **PKY2176** | PKY974, pCU305::*LEU*, pCU306::*URA3* | This study |
| **PKY2173** | PKY2023, pCU305::*LEU*, pCU306::*URA3* | This study |
| **PKY2172** | PKY2023, pCU305::*LEU*, PKP2 pCU306::*URA3* | This study |
| **PKY2171** | PKY2023, PKP1 pCU305::*LEU*, pCU306::*URA3* | This study |
| **PKY2170** | PKY2023, PKP1 pCU305::*LEU*, PKP2 pCU306::*URA3* | This study |
| **PKY520** | TVY1, *arg4Δ:: G418 ^R^* | This study |
| **PKY2154** | PKY520, mtDHFR-RFP pCU306::*URA3* | This study |
| **PKY2196** | PKY2154, *pda1Δ::HIS5 S.p* | This study |
| **HAY809** | HAY75, *aup1Δ::HIS5 S.p.* | Kolitsida et al, *PNAS,* 2019 |
| **PKY2088** | HAY809, Mdh1-GFP::G418 ^R^ | This study |
| **PKY2204** | PKY2088, *pda1Δ::* NAT*^R^* | This study |
| **PKY2077** | HAY75, *lat1Δ::HIS5 S.p.* | This study |
| **PKY2083** | PKY2077, MDH1-GFP::*G418 ^R^* | This study |
| **PKY15** | *ptc5Δ::* NAT*^R^* | Kolitsida et al, *PNAS,* 2019 |
| **PKY2239** | PKY15, MDH1-GFP::*G418 ^R^* | This study |
| **PKY835** | *ptc7Δ::HIS5 S.p.* | This study |
| **PKY2234** | PKY835, MDH1-GFP::*G418 ^R^* | This study |
| **PKY2250** | PKY835, *pda1Δ::G418 ^R^* : *URA3* | This study |
| **PKY2251** | PKY2250, MDH1-GFP:: NAT*^R^* | This study |
| **PKY2252** | PKY15, *pda1Δ::HIS5 S.p* | This study |
| **PKY2284** | PKY2252, *ptc7Δ:: DP* | This study |
| **PKY2286** | PKY2284, MDH1-GFP::*G418 ^R^* | This study |
| **PKY2298** | PKY2205, *lat1Δ::HIS5 S.p* | This study |
| **PKY2316** | PKY974, mtRFP pCU306::*URA3* | This study |
| **PKY2319** | PKY2023, mtRFP pCU306::*URA3* | This study |
| **PKY723** | *mdh1Δ::* NAT*^R^* | This study |
| **PKY2357** | PKY723, *pda1Δ::HIS5 S.p* | This study |
| **PKY1086** | TVY1, *pda1Δ::G418 ^R^* : *URA3* | This study |
| **PKY1078** | TVY1, PKP1-HA:*:HIS5 S.p* | This study |
| **PKY2434** | PKY1086, PKP1-HA:*:HIS5 S.p* | This study |
| **PKY881** | TVY1, PKP2-HA:*:HIS5 S.p* | This study |
| **PKY2223** | PKY1086, PKP2-HA:*:HIS5 S.p* | This study |
| **PKY2237** | TVY1, AUP1-HA:*:HIS5 S.p* | This study |
| **PKY2138** | PKY1086, AUP1-HA:*:HIS5 S.p* | This study |
| **PKY2257** | PKY2202, ACO1-GFP::*G418 ^R^* | This study |
| **PKY2258** | PKY2206, ACO1-GFP::*G418 ^R^* | This study |
| **PKY2255** | PKY2202, QCR2-GFP::*G418 ^R^* | This study |
| **PKY2256** | PKY2206, QCR2-GFP::*G418 ^R^* | This study |
| **PKY2474** | PKY2202, *lat1Δ*:: *G418 ^R^* | This study |
| **PKY2498** | PKY2474, PDA1-FLAG pRS305::LEU | This study |
