# Supplementary material for: The pyruvate dehydrogenase complex regulates mitophagic trafficking and protein phosphorylation
Source: Life Sci Alliance. 2023 Jul 13;6(9):e202302149. doi: 10.26508/lsa.202302149 (PMC10345312; doi:10.26508/lsa.202302149)
Supplement: Supplementary file 3 [file LSA-2023-02149_TableS3.doc]

**Supplementary Table 3. Oligonucleotides used in this study**

| **Primers** | **Purpose** | **Primers** |
| --- | --- | --- |
| **P1** | Amplification of mtDHFR-RFP for the construction of pPKB128 | Forward: 5' ATACTAGAATTCTACAACATGGCCTCCACTCGTGTC |
| **P2** |  | Reverse: 5' ATACTA AGATCT GTCTTTCTTCTCGTAGAC |
| **P3** | Amplification of PKP1 for the construction of pPKB132 | Forward: 5' ATACTAACTAGTTACAACATGTGGAAGATTATGCGTTC |
| **P4** |  | Reverse: 5' ATACTAATCGATTTACTTTTTGGAGAGTAGTG |
| **P5** | Amplification of PKP2 for the construction of pPKB90 | Forward: 5' ATACTAGGATCCTACAACATGTCTAAGTATCAAATTAA |
| **P6** |  | Reverse: 5' ATACTAATCGATTCACACTTTATCTAATTGTAG |
| **P7** | Amplification of DHFR for the construction of pKB115 | Forward: 5' ATACTAGGATCCTACAACATGATCAGTCTGATTGCGG |
| **P8** |  | Reverse: 5' ATACTAAGATCTCCGCCGCTCCAGAATCTC |
| **P9** | Amplification of PDA1 for the construction of pPKB118 | Forward: 5' ATACTAACTAGTTACAACATGCTTGCTGCTTCATTC |
| **P10** |  | Reverse: 5' ATACTAATCGATTTAATCCCTAGAGGCAAAAC |
| **P11** | Amplification of LAT1 for the construction of pPKB145 | Forward: 5' ATACTACTGCAGCTGTTTTCCAAGATATAC |
| **P12** |  | Reverse: 5' ATACTAGTCGACGTAAGTTCAAAAAGTTTG |
| **P13** | Amplification of PKP1 for the construction of PKB151 | Forward: 5' ATACTAACTAGTTACAACATGTGGAAGATTATGCGTTC |
| **P14** |  | Reverse: 5' ATACTACTCGAGTTACTTTTTGGAGAGTAGTG |
| **P15** | Amplification of PDA1-FLAG for the construction of PKB176 | Forward: 5' ATACTAACTAGTGAGTTGATTTGTTCGTAC |
| **P16** |  | Reverse: 5' ATACTACTCGAGTTACTTGTCATCGTCATCTTTA |
| **D1** | Amplification of  *His5 S.p.*  cassette for the deletion of *pda1Δ* | Forward: 5' GTTGGATACAGCAATAAGAAAGGAAACCACATTTGTGCCA CCCGGGCTGCAGGAATTC |
| **D2** |  | Reverse: 5' CGATCACAGCACTATTATTTTATTTTTCCTTACGATTTAA TCGACGGTATCGATAAGC |
| **D3** | Amplification natMX4 cassette for the deletion of *pep4Δ* | Forward: 5' ATTTAATCCAAATAAAATTCAAACAAAAACCAAAACTAAC CAGCTGAAGCTTCGTACG |
| **D4** |  | Reverse: 5' TATTGTTATCTACTTATAAAAGCTCTCTAGATGGCAGAAA CATAGGCCACTAGTGGATCTG |
| **D5** | Amplification of natMX4 cassette for the deletion of *pda1Δ* | Forward: 5' GTTGGATACAGCAATAAGAAAGGAAACCACATTTGTGCCACAG CTGAAGCTTCGTACG |
| **D6** |  | Reverse: 5' CGATCACAGCACTATTATTTTATTTTTCCTTACGATTTAA CATAGGCCACTAGTGGATCTG |
| **D7** | Amplification of  *G418^R^* cassette for the deletion of *ptc7Δ* | Forward:5'ATAAAAGCGGTCCAGAAAACAAACGACAAAGCCACCAAAAGAGCTCGTTTTCGACACTGG |
| **D8** |  | Reverse:5'TTTATTTACACTGCTTTCCAGGAGATTAAAGAGCGGAGTGTCCTTACCATTAAGTTGATC |
| **D9** | Amplification of  *His5 S.p.*  cassette for the deletion of  *lat1Δ* | Forward:5' GTAAACTGCATCCAGTAAAATTAATAGTTATTAGCGTACT CCCGGGCTGCAGGAATTC |
| **D10** |  | Reverse: 5' AGATACGCATTTACTGGCGAATTTTATTTTCATTCTAACC TCGACGGTATCGATAAGC |
| **D11** | Amplification of  *G418^R^*  cassette for the deletion of *arg4Δ* | Forward:5'GCTCAAAAGCAGGTAACTATATATAACAAGACTAAGGCGGTGGTCTTGCGCAACACG |
| **D12** |  | Reverse:5'CTAATTTAATTGGGATTTCAAATTATCCAATTGCTTCAATGAATTCGAGCTCGTTTAAAC |
| **D13** | Amplification of  *His5 S.p.*  cassette for the deletion of *mpc1Δ* | Forward:5' ATATATACGTATAGATTTTATTGCACTGTGATCAAAAAGA CCCGGGCTGCAGGAATTC |
| **D14** |  | Reverse:5' TCCATCTAGTCACCTACTTCAGGTTCTTAGACTGCTCGTT TCGACGGTATCGATAAGC |
| **D15** | Amplification of natMX4 cassette for the deletion of *ptc5Δ* | Forward:5' TTTCAACAGAAGAAGTGCTTTTACTTCTCT CAATCTCTCC3' |
| **D16** |  | Reverse:5' ATCCTCTGGTATATACCTACCTCAGCATAAGTTTATATC |
| **D17** | Amplification of  *His5 S.p.*  cassette for the deletion of *ptc7Δ* | Forward:5' ATAAAAGCGGTCCAGAAAACAAACGACAAAGCCACCAAAA |
| **D18** |  | Reverse:5'TTATTTACACTGCTTTCCAGGAGATTAAAGAGCGGAGTG |
| **D19** | Amplification of natMX4 cassette for the deletion of *mdh1Δ* | Forward:5' AAGAAAAAAAACAAAAGGAAAAGGAAGGATACCATATACA CAGCTGAAGCTTCGTACG |
| **D20** |  | Reverse:5'TTTTTCCCTATTTTTCACTCTATTTCTGATCTTGAACAATCATAGGCCACTAGTGGATCTG |
| **M1** | Site Directed Mutagenesis of T313A on Pda1 | Forward:5' GGTACGGTGGCCATGCTATGTCTGATCCC |
| **M2** |  | Reverse:5' GGGATCAGACATAGCATGGCCACCGTACC |
| **M3** | Site Directed Mutagenesis of R322Cys on Pda1 | Forward:5' CCGGTACTACCTACTGAACTAGAGACGAG |
| **M4** |  | Reverse:5' CTCGTCTCTAGTTCAGTAGGTAGTACCGG |
| **M5** | Site Directed Mutagenesis of K75R on Lat1 | Forward:5' CGAAATAGAAACAGACTCTGCTCAAATGGACTTTG |
| **M6** |  | Reverse:5' CAAAGTCCATTTGAGCAGAGTCTGTTTCTATTTCG |
