# Supplementary material for: The pyruvate dehydrogenase complex regulates mitophagic trafficking and protein phosphorylation
Source: Life Sci Alliance. 2023 Jul 13;6(9):e202302149. doi: 10.26508/lsa.202302149 (PMC10345312; doi:10.26508/lsa.202302149)
Supplement: Supplementary file 4 [file LSA-2023-02149_TableS4.doc]

**Supplementary Table 4. Plasmids used in this study**

| **Plasmid** | **Description** | **Backbone vector** | **RE sites in backbone vector/insert** | **Yeast Markers** | **Insert** | **Cloning Primers** | **Reference/ source** |
| --- | --- | --- | --- | --- | --- | --- | --- |
| **pCU416** | **-** | - | *URA3* | **-** | **-** | **-** | (Labbe S et al.., 1999) |
| **pRS415** | **-** | **-** | *LEU2* | **-** | **-** | **-** | (Sikorsky R et al., 1989**)** |
| **pCU306** | **-** | **-** | *URA3* | **-** | **-** | **-** | (Sikorsky R et al., 1989**)** |
| **pCU305** | **-** | **-** | *LEU2* | **-** | **-** | **-** | (Sikorsky R et al., 1989**)** |
| **pPKB78** | pCU416-PDA1 | pCU416 | ClaI, SpeI | *URA3* | PDA1 | P11,P12 | This study |
| **pPKB84** | pCU416-*PDA1^S313A^* | pCU416 | ClaI, SpeI | *URA3* | PDA1 *^S313A^* | M1, M2 | This study |
| **pPKB89** | pCU416-PKP1 | pCU416 | ClaI, SpeI | *URA3* | PKP1 | - | Kolitsida et al, *PNAS,* 2019 |
| **pPKB132** | pCU305-PKP1 | pCU305 | ClaI, SpeI | *LEU2* | PKP1 | P5,P6 | This study |
| **pPKB90** | pCU416-PKP2 | pCU416 | ClaI, SpeI | *URA3* | PKP2 | - | Kolitsida et al, *PNAS,* 2019 |
| **pPKB133** | pCU306-PKP2 | pCU306 | ClaI, SpeI | *URA3* | PKP2 | P7, P8 | This study |
| **pKB185** | pYX142  mt-DHFR-GFP | pYX142 | BamHI, BglII | *LEU2* | mt-DHFR-GFP | P9, P10 | This study |
| **PKB128** | pCU306  mt-DHFR-RFP | pCU416 | ECORI, BglII | *URA3* | mt-DHFR-RFP | P3, P4 | This study |
| **PKB145** | pRS414 LAT1 | pRS414 | PstI, SalI | *TRP* | LAT1 | P13, P14 | This study |
| **PKB148** | pRS414 LAT1^K75R^ | pRS414 | PstI, SalI | *TRP* | LAT1 ^K75R^ | M5, M6 | This study |
| **PKB124** | pCU416-*PDA1^R322Cys^* | pCU416 | ClaI, SpeI | *URA3* | PDA1 *^R322Cys^* | M3, M4 | This study |
| **PKB113** | pCU306  mt-RFP | pCU306 | ECORI, XhoI | *URA3* | mt-RFP | - | This study |
| **PKB151** | pCU415-PKP1 | pCU415 | XhoI, SpeI | *LEU2* | PKP1 | P15, P16 | This study |
| **pPKB24** | pRS415- MDH1^T199A^-GFP | pRS415 | BamHI, SacI | *LEU2* | [MDH1p]-  MDH1^T199A^-GFP | - | Kolitsida et al, *PNAS,* 2019 |
| **pPKB40** | pRS415- MDH1^T199D^-GFP | pRS415 | BamHI, SacI | *LEU2* | [MDH1p]-  MDH1^T199D^-GFP | - | Kolitsida et al, *PNAS,* 2019 |
| **pPKB65** | pRS415-MDH1-GFP | pRS415 | BamHI, SacI | *LEU2* | [MDH1p]-  MDH1-GFP | - | Kolitsida et al, *PNAS,* 2019 |
| **PKB176** | pRS305-PDA1-FLAG | pRS415 | XhoI, SpeI | *LEU2* | [PDA1p]-  PDA1-FLAG | P15,P16 | This study |
| **PKB138** | pCU416-  AUP1-HA | pCU416 | ClaI, SpeI | *URA3* | AUP1 | - | Kolitsida et al, *PNAS,* 2019 |
