# Supplementary material for: Correction: The pyruvate dehydrogenase complex regulates mitophagic trafficking and protein phosphorylation
Source: Life Sci Alliance. 2024 Mar 14;7(6):e202402684. doi: 10.26508/lsa.202402684 (PMC10940167; doi:10.26508/lsa.202402684)
Supplement: Reviewer comments [file LSA-2024-02684_review_history.pdf]

Correction: The pyruvate dehydrogenase complex regulates mitophagic trafficking and protein phosphorylation

Panagiota Kolitsida, Vladimir Nolic, Jianwen Zhou, Michael Stumpe, Natalie Niemi, Jörn Dengjel, and Hagai Abeliovich
DOI: https://doi.org/10.26508/lsa.202402684

Corresponding author(s): Hagai Abeliovich, Hebrew University of Jerusalem

**Review Timeline:**	Submission Date:	2024-02-28
	Accepted:	2024-03-01

Scientific Editor: Eric Sawey, PhD

Transaction Report:

(Note: With the exception of the correction of typographical or spelling errors that could be a source of ambiguity, letters and reports are not edited. The original formatting of letters and referee reports may not be reflected in this compilation.)

March 1, 2024

RE: Life Science Alliance Manuscript #LSA-2024-02684

Prof. Hagai Abeliovich
Hebrew University of Jerusalem
Dept. of Biochemistry and Food Science
Hebrew University of Jeruslaem
Rehovot, Israel 76100
Israel

Dear Dr. Abeliovich,

Thank you for submitting your Correction entitled "Correction: The pyruvate dehydrogenase complex regulates mitophagic trafficking and protein phosphorylation".

Your correction will now progress through copyediting and proofing.

IMPORTANT: If you will be unreachable at any time, please provide us with the email address of an alternate author. Failure to respond to routine queries may lead to unavoidable delays in publication.

You can contact the journal office with any questions, contact@life-science-alliance.org

Sincerely,

Eric Sawey, PhD
Executive Editor
Life Science Alliance
http://www.lsajournal.org

March 1, 2024

RE: Life Science Alliance Manuscript #LSA-2024-02684

Prof. Hagai Abeliovich
Hebrew University of Jerusalem
Dept. of Biochemistry and Food Science
Hebrew University of Jeruslaem
Rehovot, Israel 76100
Israel

Dear Dr. Abeliovich,

Thank you for submitting your Correction entitled "Correction: The pyruvate dehydrogenase complex regulates mitophagic trafficking and protein phosphorylation".

Your correction will now progress through copyediting and proofing.

IMPORTANT: If you will be unreachable at any time, please provide us with the email address of an alternate author. Failure to respond to routine queries may lead to unavoidable delays in publication.

You can contact the journal office with any questions, contact@life-science-alliance.org

Sincerely,

Eric Sawey, PhD
Executive Editor
Life Science Alliance
http://www.lsajournal.org
